# Association between body fat decrease during the first year after diagnosis and the prognosis of idiopathic pulmonary fibrosis: CT-based body composition analysis

**DOI:** 10.1186/s12931-024-02712-6

**Published:** 2024-02-28

**Authors:** Ji Young Lee, Soon Ho Yoon, Jin Mo Goo, Jimyung Park, Jong Hyuk Lee

**Affiliations:** 1 Department of Radiology, Seoul National University Hospital, Seoul National University College of Medicine, 101, Daehak-ro, Jongno-gu, Seoul, 03080 Republic of Korea; 2 Institute of Radiation Medicine, Seoul National University Medical Research Center, 101, Daehak-ro, Jongno-gu, Seoul, 03080 Korea; 3 Cancer Research Institute, Seoul National University, 101, Daehak-ro, Jongno-gu, Seoul, 03080 Korea; 4 Division of Pulmonary and Critical Care Medicine, Department of Internal Medicine, Seoul National University Hospital, 101, Daehak-ro, Jongno- gu, Seoul, 03080 Republic of Korea

**Keywords:** Idiopathic pulmonary fibrosis, Prognosis, Body composition, Body fat, Deep learning

## Abstract

**Background:**

The prognostic role of changes in body fat in patients with idiopathic pulmonary fibrosis (IPF) remains underexplored. We investigated the association between changes in body fat during the first year post-diagnosis and outcomes in patients with IPF.

**Methods:**

This single-center, retrospective study included IPF patients with chest CT scan and pulmonary function test (PFT) at diagnosis and a one-year follow-up between January 2010 and December 2020. The fat area (cm^2^, sum of subcutaneous and visceral fat) and muscle area (cm^2^) at the T12-L1 level were obtained from chest CT images using a fully automatic deep learning-based software. Changes in the body composition were dichotomized using thresholds dividing the lowest quartile and others, respectively (fat area: -52.3 cm^2^, muscle area: -7.4 cm^2^). Multivariable Cox regression analyses adjusted for PFT result and IPF extent on CT images and the log-rank test were performed to assess the association between the fat area change during the first year post-diagnosis and the composite outcome of death or lung transplantation.

**Results:**

In total, 307 IPF patients (69.3 ± 8.1 years; 238 men) were included. During the first year post-diagnosis, fat area, muscle area, and body mass index (BMI) changed by -15.4 cm^2^, -1 cm^2^, and − 0.4 kg/m^2^, respectively. During a median follow-up of 47 months, 146 patients had the composite outcome (47.6%). In Cox regression analyses, a change in the fat area < -52.3 cm^2^ was associated with composite outcome incidence in models adjusted with baseline clinical variables (hazard ratio [HR], 1.566, *P* = .022; HR, 1.503, *P* = .036 in a model including gender, age, and physiology [GAP] index). This prognostic value was consistent when adjusted with one-year changes in clinical variables (HR, 1.495; *P* = .030). However, the change in BMI during the first year was not a significant prognostic factor (*P* = .941). Patients with a change in fat area exceeding this threshold experienced the composite outcome more frequently than their counterparts (58.4% vs. 43.9%; *P* = .007).

**Conclusion:**

A ≥ 52.3 cm^2^ decrease in fat area, automatically measured using deep learning technique, at T12-L1 in one year post-diagnosis was an independent poor prognostic factor in IPF patients.

**Supplementary Information:**

The online version contains supplementary material available at 10.1186/s12931-024-02712-6.

## Background

Idiopathic pulmonary fibrosis (IPF) is a chronic, progressive idiopathic interstitial lung disease characterized by worsening respiratory symptoms, physiological impairment, and eventual death due to respiratory failure or its comorbidities [1]. Most IPF patients have progressive disease courses, with their median survival ranging from 2.5 to 3.5 years [2, 3]. Therefore, physicians need noninvasive and reproducible indicators to predict the prognosis, including forced vital capacity (FVC); diffusing capacity of carbon monoxide (DL_CO_); the gender, age, and physiology (GAP) index; and respiratory hospitalization [4–10].

During the first year after diagnosis, approximately 15–20% of IPF patients lose more than 5% of their weight [11, 12]. An increase in caloric requirements due to the increased work of breathing and decreased appetite by side-effects of antifibrotics, persistent cough, or psychosocial issues such as depression are presumed to cause unintended weight loss in IPF patients [13]. Indeed, studies have reported that low baseline body mass index (BMI) [14–17] or a decrease in body weight and BMI during the disease course [11, 12, 14, 15, 18–22] can adversely affect the prognosis of patients with IPF. However, these somatotype indicators only reflect the total weight (i.e., a sum of body fat, muscle, bone, and organs), not the body composition. Therefore, the prognostic role of body composition in patients with IPF remained underexplored, particularly in relation to body fat.

Recent advances in deep learning techniques have improved the quantification of body composition using diagnostic images such as computed tomography (CT). This approach can directly quantify each body component (e.g., fat or muscle) objectively and reproducibly [23, 24]. This study investigated the changes in body fat measured in chest CT images during the first year post-diagnosis using deep learning-based body composition analyses, and assessed the association between these changes and outcomes in patients with IPF.

## Methods

This retrospective study was approved by the Institutional Review Board of Seoul National University Hospital, and the requirement for written informed consent was waived (IRB No.: 2208-165-1354).

### Study sample

This single-center, cohort study included consecutive patients diagnosed with IPF between January 2010 and December 2020 at a tertiary hospital. IPF was diagnosed through multi-disciplinary team discussions, following the integration of clinical, radiological, and histological data when available. Among the IPF patients, we excluded patients without either baseline (i.e., at diagnosis) or one-year follow-up pulmonary function test (PFT) results and chest CT images. Baseline and one-year follow-up PFT and chest CT were defined with a buffer of 3 additional months to account for variability in patients’ follow-up strategy (i.e., examinations of 3 months before and after diagnosis were defined as baseline examinations; examinations within 9 and 15 months after diagnosis were defined as 1-year follow-up examinations). Given that our patient cohort consisted of individuals whose IPF diagnosis was established at our center, it was ensured that they maintained a stable disease course during the first year after diagnosis.

### Clinical-radiological variables

Clinical data, including age, sex, BMI, smoking status, GAP index, use of antifibrotic agents, and PFT results (FVC and DL_CO_) were obtained from the electronic medical records. BMI was categorized as underweight (< 18.5 kg/m^2^), normal (18.5–24.9 kg/m^2^), overweight (25-29.9 kg/m^2^), and obese (> 30 kg/m^2^) [25]. Given the prognostic value of FVC and DL_CO_ [26–28], the thresholds for significant declines in FVC and DL_CO_ were defined as absolute declines of 5% and 10% from the baseline results, respectively. On the baseline chest CT images, one thoracic radiologist (J.Y.L. with 5 years of experience in thoracic images) measured the pulmonary artery diameter in the axial plane at the bifurcation, orthogonal to the long axis of the pulmonary artery [29, 30].

Commercially available deep learning-based texture analysis software (AVIEW Lung Texture version 1.1.43.7, Coreline Soft) was used to quantify IPF disease extent (performed by one radiologist [J.H.L.] with 11 years of experience in body imaging). This tool performs fully automatic segmentation of the total lung parenchyma and each finding (ground-glass opacity, reticular opacity, honeycombing cyst, emphysema, and consolidation) on CT images and provides quantification results as a percentage (%) of the total lung volume. IPF disease extent was defined as the sum of ground-glass opacity, reticular opacity, and honeycombing cysts.

### CT examination

CT scans were acquired with 11 CT scanners (Brilliance 64, Ingenuity, ICT 256, Phillips Medical Systems, Best, the Netherlands; Sensation 16, Somatom Definition, Somtom Forse, Iqon Spectral CT, Siemens Medical Solutions, Forchheim, Germany; Aquilion One, Toshiba, Tokyo, Japan; Discovery CT750 HD, LightSpeed Ultra, Revolution, GE Medical Systems, Waukesha, Wis). The detailed parameters for CT acquisition were as follows: tube voltage, 100–120 kVp; tube current, standard (reference mAs, 60–120) to low-dose (reference mAs, 30) with automatic exposure control; slice thickness, 0.75-3.0 mm; reconstruction interval, 1.0–3.0 mm; and standard or sharp reconstruction kernel. All CT image data were evaluated regardless of intravenous contrast medium use. In contrast-enhanced CT scans, post-contrast CT images were obtained 60 s after the injection of 90 mL of iodinated contrast medium (iopamidol; iodine content, 300 mg/mL) at 3 mL/s, followed by 30 mL of saline chaser at the same rate.

### Body composition analysis

Baseline and one-year follow-up CT images were imported into commercially available deep learning-based body composition analysis software (DEEPCATCH, v1.1.8.0, MEDICALIP Co. Ltd.). One radiologist (J.H.L.) confirmed the completeness of the software’s segmentation results. The software calculated CT-derived parameters, including subcutaneous fat area (cm^2^), visceral fat area (cm^2^), and muscle area (cm^2^) at T12-L1. Specifically, volumes of subcutaneous fat, visceral fat, and muscle at T12-L1 were captured and then normalized by dividing by the height of the T12-L1 vertebral bodies, and these values were defined as body composition areas, respectively. We used this measurement approach instead of using whole-body composition results obtained on chest CT because not all patients had exactly the same CT scan coverage. Indeed, multi-slice averaged body composition analysis results at the level of T12-L1 are known to be well correlated with whole-body composition [31]. Therefore, in this study, the subcutaneous and visceral fat areas were summed and defined as body fat area to represent whole-body fat. The fat areas and muscle areas from baseline and one-year follow-up CT images and their differences were obtained.

### Outcomes

The outcome of this study was the occurrence of death or lung transplantation, defined as a composite outcome. Survival status and date of death were acquired from a database of the Ministry of the Interior and Safety, Korea, and information on lung transplantation was obtained from electronic medical records. Survival time was censored on March 31, 2023. For individuals who died or underwent lung transplantation, the time of censoring was defined as the date of death or transplantation, respectively.

### Statistical analysis

Continuous variables are presented as mean values with standard deviations or median values with interquartile ranges according to the normality of the data distribution.

Spearman correlation coefficients were used to evaluate the relationship of changes in the fat or muscle area with changes in weight, BMI, and PFT results.

To analyze the prognostic value of change in the fat area, univariable and multivariable Cox regression analyses were performed. For multivariable regression analyses, variables with a *P*-value < 0.2 in the univariable analysis and BMI (both results at baseline and change in follow-up) were included. BMI was used despite being non-significant in the univariable analysis because it has been reported as a significant prognostic factor in IPF patients [12, 14–17, 19]. To robustly evaluate the prognostic value of change in the fat area, we separately performed multivariable regression analyses with the following variables: (a) baseline clinical-radiological variables, (b) baseline clinical-radiological variables including the GAP score [32], and (c) variables obtained at one-year follow-up visits, such as a change in BMI or PFT results. As there are no established optimal cut-off values for body composition in IPF patients, we categorized them into either the lowest quartile or the upper three quartiles based on their baseline body composition and the change during the first year, respectively. This approach derived from assessing the association between change in the fat area during the first year after diagnosis and each of event (composite outcome and death), using natural cubic splines in a Cox regression model (Appendix S1 and Fig S1).

A Kaplan-Meier plot was drawn, and the log-rank test was performed according to whether patients were categorized in the lowest quartile of fat area change. As a sensitivity analysis, the above-described survival analyses were performed for the outcome of death alone.

To assess the effect of baseline BMI on the prognostic value of fat area change, we performed subgroup analyses in IPF patients whose baseline BMI was < 25 kg/m^2^ and ≥ 25 kg/m^2^ separately. We set the thresholds to define the lowest quartile of fat change in each group, respectively.

All statistical analyses were performed using SPSS version 21.0 (IBM Corp.) and SAS version 9.4 (SAS Institute Inc.), and a *P*-value of < 0.05 was considered to indicate statistical significance.

## Results

### Baseline characteristics

**Fig. 1:**
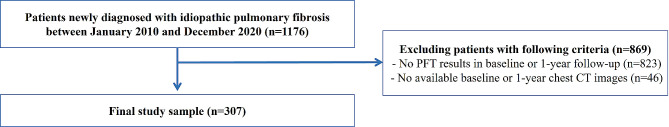
Flow diagram showing the study sample

A total of 307 individuals (238 men and 69 women; mean age, 69 ± 8.1 years) were included in this study (Fig. 1). The baseline characteristics of this study sample are described in Table 1. At baseline, the mean BMI was 24.5 ± 3.0 kg/m^2^ (underweight, *n* = 6 [2.0%]; normal, *n* = 179 [58.3%]; overweight, *n* = 108 [35.2%]; obese, *n* = 14 [4.6%]). Furthermore, 209 (68.1%) patients were ever smokers. The baseline FVC and DL_CO_ were 80.8 ± 16.5% and 64.9 ± 17.9% predicted, respectively. The median baseline GAP score was 3 (interquartile range [IQR], 2–4), and the median total IPF extent was 12.3% (IQR, 7.6–18.3%).

**Table 1:** Baseline characteristics of the study sample

Variables	Study sample (*n* = 307)
Age (years)	69.3 ± 8.1
Sex	
Male	238 (77.5%)
Female	69 (22.5%)
Baseline body mass index (kg/m^2^)	24.5 ± 3.0
<18.5, underweight	6 (2.0%)
18.5–25, normal	179 (58.3%)
25–30, overweight	108 (35.2%)
>30, obese	14 (4.6%)
Body mass index at 1-year follow-up (kg/m^2^)	24.1 ± 3.0
Smoking status	
Never smoker	98 (31.9%)
Ever smoker	209 (68.1%)
Baseline FVC (% predicted)	80.8 ± 16.5
Baseline DL_CO_ (% predicted)	64.9 ± 17.9
FVC decline ≥ 5% (% predicted)	93 (30.3%)
DL_CO_ decline ≥ 10% (% predicted)	59 (19.2%)
GAP index score (IQR)	3 (2–4)
Antifibrotic agents	
No use	73 (23.8%)
Use	234 (76.2%)
Pulmonary artery diameter (mm) (IQR)	29 (26–31)
Total IPF extent at baseline CT images (%) (IQR)	12.3 (7.6–18.3)
Fat area at T12-L1 on baseline CT (cm^2^) (IQR)	191.4 (136.7-255.8)
Muscle area at T12-L1 on baseline CT (cm^2^) (IQR)	100.8 (84.7-112.5)
Fat area at T12-L1 at the one-year follow-up CT (cm^2^) (IQR)	165.0 (115.5-226.5)
Muscle area at T12-L1 at the one-year follow-up CT (cm^2^) (IQR)	100.7 (83.5-113.8)
Interval between pulmonary function test (months) (IQR)	12 (11–13)
Interval between CT scans (months) (IQR)	12 (11–14)
Follow-up duration (months) (IQR)	47 (33–64)
Composite outcome*	146 (47.6%)
Lung transplantation	15 (4.9%)
Death	139 (45.3%)

FVC: forced vital capacity; DL_CO_: diffusing capacity of carbon monoxide; GAP: gender, age, and physiologic variables; IQR: interquartile range; IPF: idiopathic pulmonary fibrosis

*Composite outcome was the occurrence of death or lung transplantation

The median intervals between PFT and CT were both 12 months (IQR: 11–13 months for PFT; 11–14 months for CT). During the first year post-diagnosis, BMI decreased by 0.4 kg/m^2^ (24.1 ± 3.0 kg/m^2^ at follow-up). Ninety-three (30.3%) and 59 (19.2%) patients had absolute declines of FVC of 5% and DL_CO_ of 10%, respectively. During a median follow-up duration of 47 months (IQR, 33–64 months), 146 patients had the composite outcomes (47.6%; death, *n* = 139, 45.3%; lung transplantation, *n* = 15, 4.9%).

### Body composition analysis results

**Fig. 2:**
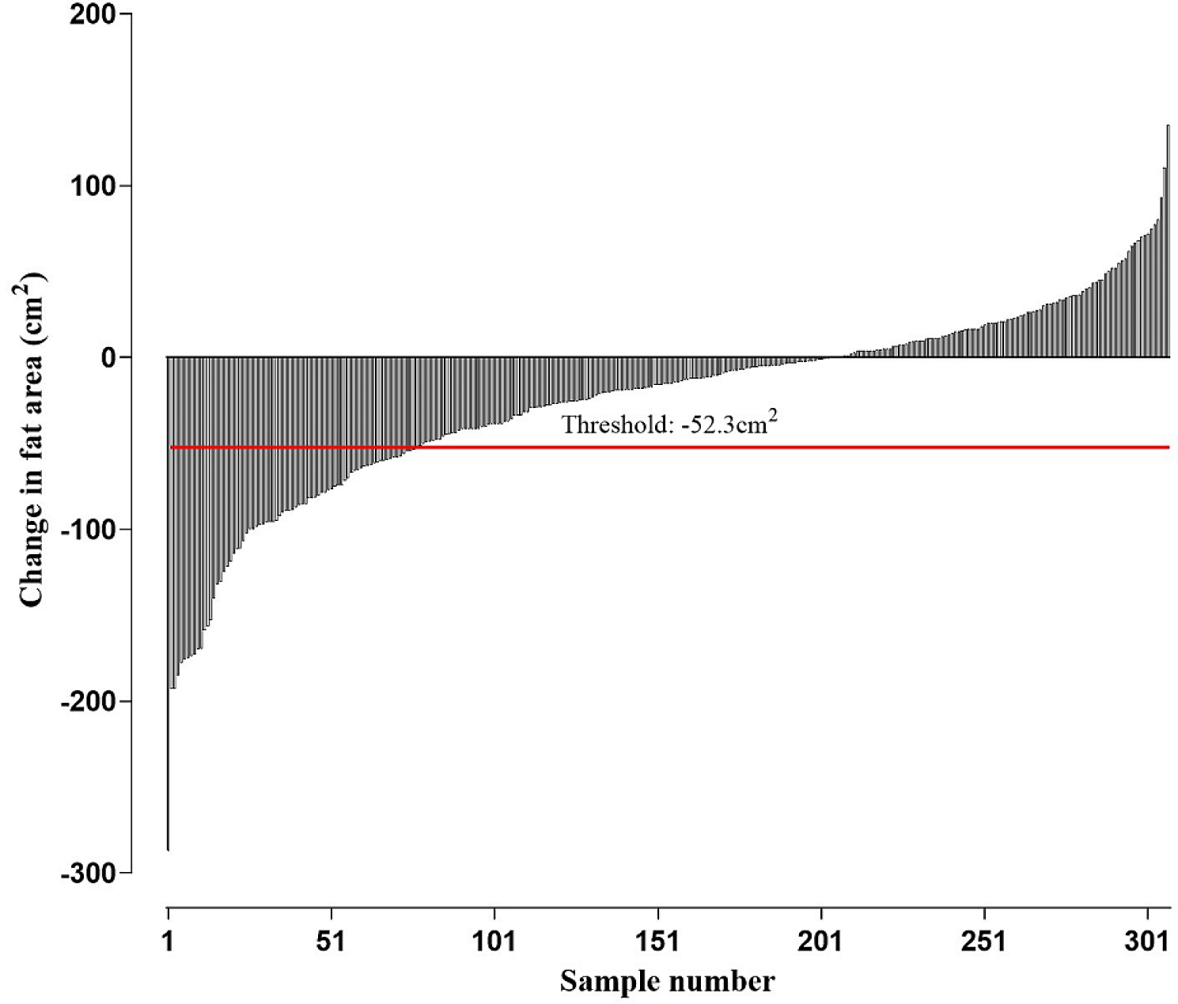
Waterfall plot for the change in the fat area during the first year post-diagnosis in the study sample. The threshold splitting the lowest quartile and others was − 52.3 cm^2^

The median baseline fat area was 191.4 cm^2^ (IQR, 136.7-255.8 cm^2^), and the median baseline muscle area was 100.8 cm^2^ (IQR, 84.7-112.5 cm^2^). At the one-year follow-up CT, the median fat and muscle areas were 165.0 cm^2^ (IQR, 115.5-226.5 cm^2^) and 100.7 cm^2^ (IQR, 83.5-113.8 cm^2^), respectively. The median changes in the fat area and muscle area were − 15.4 cm^2^ (IQR, -52.3 to 9.9 cm^2^) and − 1.0 cm^2^ (IQR, -7.4 to 6.1 cm^2^), respectively. Therefore, the thresholds splitting patients into the lowest quartile and others were − 52.3 cm^2^ for fat area change and − 7.4 cm^2^ for muscle area change, respectively (Fig. 2).

### Relationship of body composition analyses with weight, BMI, and PFT results

A change in the fat area during the first year post-diagnosis was positively correlated with weight change (*r* = .328; *P* < .001), BMI change (*r* = .335; *P* < .001) (Fig S2), and FVC decline (*r* = .122; *P* = .032). DL_CO_ showed no significant correlation with change in the fat area (*r* = .036; *P* = .527). A change in muscle area was correlated with weight change (*r* = 314; *P* < .001) and BMI change (*r* = .301; *P* < .001), but not with FVC decline (*r*=-.022; *P* = .704) and DL_CO_ decline (*r* = .008; *P* = .887) (Table 2).

**Table 2:** Spearman correlation analysis of changes in CT-derived parameters with changes in weight, body mass index (BMI), and pulmonary function test results

	Weight change		BMI change		FVC decline (% predicted)		DL_CO_ decline (% predicted)	
	r	*P* value	r	*P* value	r	*P* value	r	*P* value
Change in the fat area at T12-L1 during the first year post-diagnosis (cm^2^)	0.328	< 0.001	0.335	< 0.001	0.122	0.032	0.036	0.527
Change in muscle area at T12-L1 during the first year post-diagnosis (cm^2^)	0.314	< 0.001	0.301	< 0.001	-0.022	0.704	0.008	0.887

r: Spearman correlation coefficients; BMI: body mass index; FVC: forced vital capacity; DL_CO_: diffusing capacity of carbon monoxide

### Survival analyses

The results of the Cox regression analyses for the composite outcomes are described in Table 3. In the univariable analyses, age, baseline GAP, baseline FVC, baseline DL_CO_, a decline of FVC of 5% or a decline of DL_CO_ of 10% during the first year post-diagnosis, baseline pulmonary artery diameter, IPF extent on baseline CT, and the decrease in the fat area were significant (all *P* values < 0.05).

**Table 3:** Univariable and multivariable Cox regression analysis for the composite outcome

	Univariable analysis		Multivariable analysis (model 1)		Multivariable analysis (model 2)		Multivariable analysis (model 3)	
Variables	Hazard ratio	*P* value	Hazard ratio	*P* value	Hazard ratio	*P* value	Hazard ratio	*P* value
Age (year)	1.027 (1.006, 1.047)	0.009	1.051 (1.029, 1.074)	< 0.001				
Sex (reference: female)	1.368 (0.921, 2.031)	0.120	1.805 (1.004, 3.244)	0.049				
BMI (kg/m^2^)	0.955 (0.901, 1.013)	0.125	0.876 (0.823, 0.933)	< 0.001	0.871 (0.816, 0.929)	< 0.001		
Change in BMI during the first year post-diagnosis (kg/m^2^)	0.922 (0.804, 1.058)	0.248					0.995 (0.863, 1.147)	0.941
Smoking history (reference: never smoker)	1.328 (0.932, 1.892)	0.117	1.203 (0.727, 1.988)	0.472	1.187 (0.818, 1.721)	0.367		
Antifibrotics (reference: no use)	1.021 (0.703, 1.482)	0.914						
Baseline GAP score	1.662 (1.418, 1.948)	< 0.001			1.570 (1.326, 1.858)	< 0.001		
Baseline FVC (% predicted)	0.979 (0.968, 0.990)	< 0.001	0.986 (0.974, 0.998)	0.027				
Baseline DL_CO_ (% predicted)	0.978 (0.968, 0.987)	< 0.001	0.975 (0.963, 0.987)	< 0.001				
FVC decline ≥ 5% during the first year post-diagnosis (% predicted) (reference: FVC decline < 5%)	2.244 (1.615, 3.118)	< 0.001					2.215 (1.582, 3.100)	< 0.001
DL_CO_ decline ≥ 10% during the first year post-diagnosis (% predicted) (reference: DL_CO_ decline < 10%)	2.261 (1.577, 3.241)	< 0.001					2.278 (1.584, 3.274)	< 0.001
Baseline pulmonary artery diameter (mm)	1.080 (1.037, 1.125)	< 0.001	1.082 (1.033, 1.133)	0.001	1.090 (1.039, 1.144)	< 0.001		
IPF extent on baseline CT (%)	1.023 (1.011, 1.034)	< 0.001	1.007 (0.992, 1.023)	0.359	1.018 (1.004, 1.032)	0.009		
Muscle area at T12-L1 on baseline CT (cm^2^) (reference: upper three quartiles)	1.092 (0.751, 1.588)	0.642						
Change in muscle area at T12-L1 during the first year post-diagnosis (cm^2^) (reference: upper three quartiles)*	1.237 (0.854, 1.792)	0.261						
Fat area at T12-L1 on baseline CT (cm^2^) (reference: upper three quartiles)	0.998 (0.685, 1.454)	0.992						
Change in the fat area at T12-L1 during the first year post-diagnosis (cm^2^) (reference: upper three quartiles)*	1.619 (1.136, 2.307)	0.008	1.566 (1.066, 2.300)	0.022	1.503 (1.026, 2.202)	0.036	1.495 (1.039, 2.151)	0.030

BMI: body mass index; GAP: gender, age, and physiologic variables; FVC: forced vital capacity; DL_CO_: diffusing capacity of carbon monoxide; IPF: idiopathic pulmonary fibrosis

Model 1 is with baseline clinical-radiological variables; Model 2 is with baseline clinical-radiological variables including GAP score; Model 3 is with variables obtained at 1-year follow-ups

The composite outcome was the occurrence of death or lung transplantation

Multivariable Cox proportional hazard regression analysis was performed with variables that had *P*-values < 0.2 in the univariable analysis and BMI change since this factor was reported as a poor prognostic factor in prior study

* The lowest quartile is a group with the highest reduction of fat or mass area

**Fig. 3:**
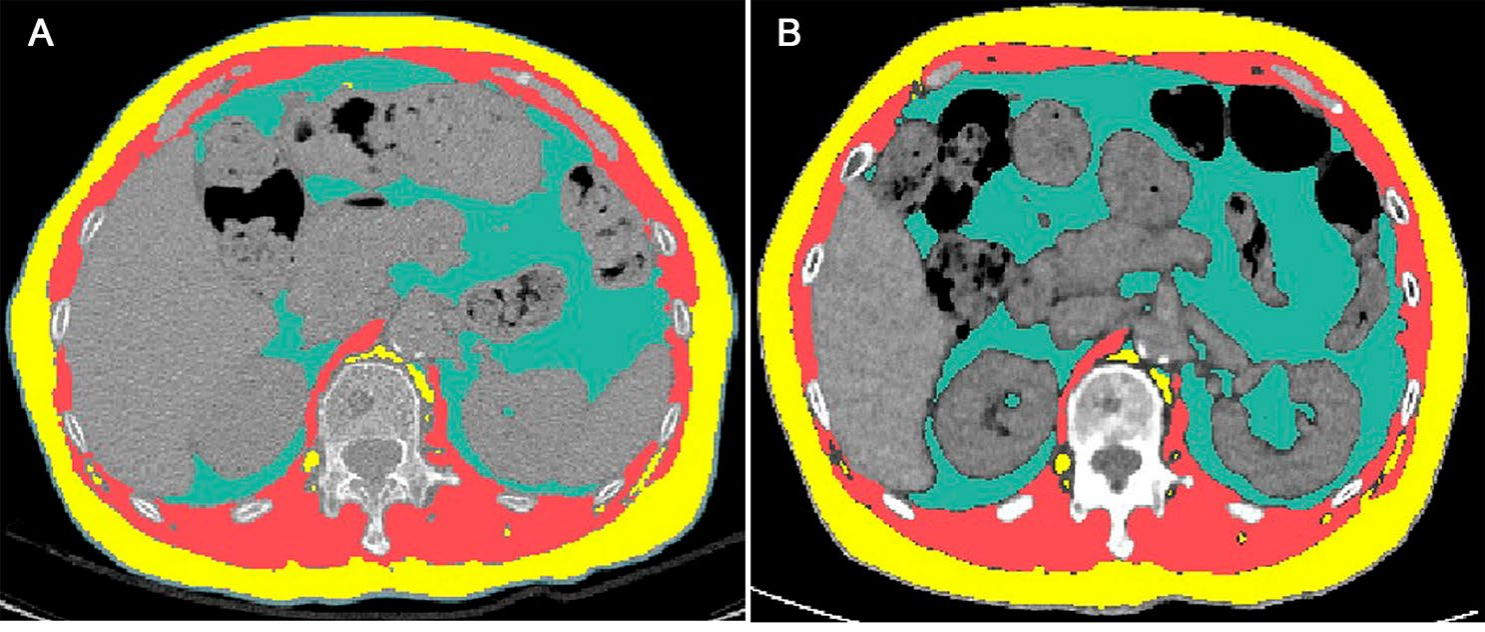
Representative case of fully automated CT-based body composition analysis in (**A**) baseline and (**B**) one-year follow-up CT images in a patient belonging to the upper three quartiles of the change in fat area. Axial images at the T12-L1 vertebral level demonstrate visceral fat (green), subcutaneous fat (yellow), and skeletal muscle (red). Between CT images, the fat area increased by 80.3 cm^2^ (baseline: 140.7 cm^2^, one-year follow-up: 221.0 cm^2^). This patient remained under follow-up for 83 months without receiving lung transplantation or dying

**Fig. 4:**
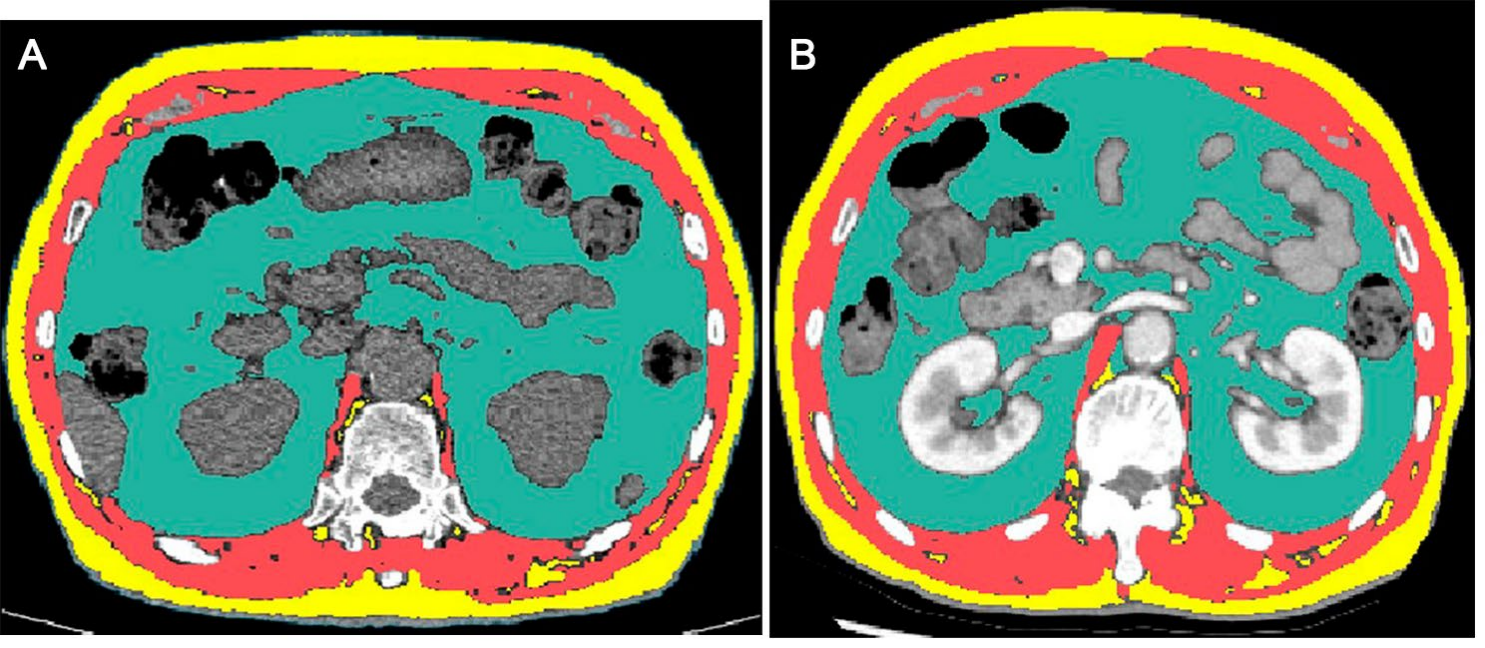
Representative case of fully automated CT-based body composition analysis in (**A**) baseline and (**B**) one-year follow-up CT images in a patient belonging to the lowest quartile of the change in fat area. Axial images at the T12-L1 vertebral level demonstrate visceral fat (green), subcutaneous fat (yellow), and skeletal muscle (red). Between CT images, the fat area decreased by 89.9 cm^2^ (baseline: 370.0 cm^2^, one-year follow-up: 280.1 cm^2^). This patient died 10 months later despite receiving a lung transplantation

After adjusting for baseline clinical-radiological variables, including age, sex, baseline PFT results, and IPF extent on baseline CT, a change of the fat area <-52.3 cm^2^ during the first year post-diagnosis was associated with the composite outcome (adjusted hazard ratio [HR]: 1.566, 95% confidence interval [CI]: 1.066, 2.300, *P* = .022). In another model in which sex, age, and PFT results were treated as GAP scores, this fat change also predicted a poor prognosis (adjusted HR: 1.503; 95% CI: 1.026, 2.202, *P* = .036). In the model with variables obtained at the one-year follow-up, including changes in BMI and PFT during the first year post-diagnosis, this threshold for fat loss maintained its prognostic value for the composite outcome (adjusted HR: 1.495; 95% CI: 1.039, 2.151; *P* = .03), but a change in BMI did not (*P* = .941). Figures 3 and 4 represent IPF cases exemplifying the prognostic values of the change in the fat area during the first year post-diagnosis. In the Cox regression analysis where body composition parameters were treated as continuous variables, the change in fat area during the first year post-diagnosis was significant in univariable analyses. However, it was not significant in the multivariable analyses (Table S1 and Table S2).

The results for death alone as an outcome are described in Table 4. A <-52.3 cm^2^ change in the fat area was also associated with death alone (adjusted HRs in all models > 1; all *P* values < 0.05).

**Table 4:** Univariable and multivariable Cox regression analysis for death

	Univariable analysis		Multivariable analysis (model 1)		Multivariable analysis (model 2)		Multivariable analysis (model 3)	
Variables	Hazard ratio	*P* value	Hazard ratio	*P* value	Hazard ratio	*P* value	Hazard ratio	*P* value
Age (year)	1.039 (1.018, 1.060)	< 0.001	1.064 (1.041, 1.088)	< 0.001				
Sex (reference: female)	1.399 (0.931, 2.102)	0.107	1.808 (0.990, 3.300)	0.054				
BMI (kg/m^2^)	0.966 (0.910, 1.024)	0.244	0.892 (0.837, 0.950)	< 0.001	0.882 (0.826, 0.942)	< 0.001		
Change in BMI during the first year post-diagnosis (kg/m^2^)	0.931 (0.809, 1.071)	0.319					1.011 (0.875, 1.170)	0.878
Smoking history (reference: never smoker)	1.311 (0.912, 1.884)	0.143	1.180 (0.706, 1.972)	0.528	1.137 (0.779, 1.660)	0.506		
Antifibrotics (reference: no use)	0.985 (0.674, 1.439)	0.937						
Baseline GAP score	1.747 (1.483, 2.058)	< 0.001			1.667 (1.401, 1.984)	< 0.001		
Baseline FVC (% predicted)	0.981 (0.970, 0.992)	0.001	0.984 (0.972, 0.997)	0.013				
Baseline DL_CO_ (% predicted)	0.981 (0.971, 0.991)	< 0.001	0.978 (0.967, 0.990)	< 0.001				
FVC decline ≥ 5% during the first year post-diagnosis (% predicted) (reference: FVC decline < 5%)	2.113 (1.507, 2.962)	< 0.001					2.070 (1.464, 2.927)	< 0.001
DL_CO_ decline ≥ 10% during the first year post-diagnosis (% predicted) (reference: DL_CO_ decline < 10%)	2.179 (1.506, 3.151)	< 0.001					2.144 (1.478, 3.109)	< 0.001
Baseline pulmonary artery diameter (mm)	1.080 (1.036, 1.126)	< 0.001	1.079 (1.028, 1.132)	0.002	1.084 (1.033, 1.139)	0.001		
IPF extent on baseline CT (%)	1.023 (1.011, 1.035)	< 0.001	1.010 (0.995, 1.026)	0.192	1.018 (1.004, 1.032)	0.014		
Muscle area at T12-L1 on baseline CT (cm^2^) (reference: upper three quartiles)	1.105 (0.752, 1.623)	0.608						
Change in muscle area at T12-L1 during the first year post-diagnosis (cm^2^) (reference: upper three quartiles)	1.144 (0.779, 1.680)	0.491						
Fat area at T12-L1 on baseline CT (cm^2^) (reference: upper three quartiles)	1.092 (0.736, 1.619)	0.659						
Change in the fat area at T12-L1 during the first year post-diagnosis (cm^2^) (reference: upper three quartiles)	1.626 (1.131, 2.337)	0.009	1.594 (1.077, 2.36)	0.020	1.513 (1.025, 2.234)	0.037	1.488 (1.021, 2.167)	0.038

BMI: body mass index; GAP: gender, age, and physiologic variables; FVC: forced vital capacity; DL_CO_: diffusing capacity of carbon monoxide; IPF: idiopathic pulmonary fibrosis

Model 1 is with baseline clinical-radiological variables; Model 2 is with baseline clinical-radiological variables including GAP score; Model 3 is with variables obtained at 1-year follow-ups

Multivariable Cox proportional hazard regression analysis was performed with variables with *P*-values < 0.2 in the univariable analysis and BMI (both results at baseline and change in follow-up) since this factor was reported as a poor prognostic factor in prior study

**Fig. 5:**
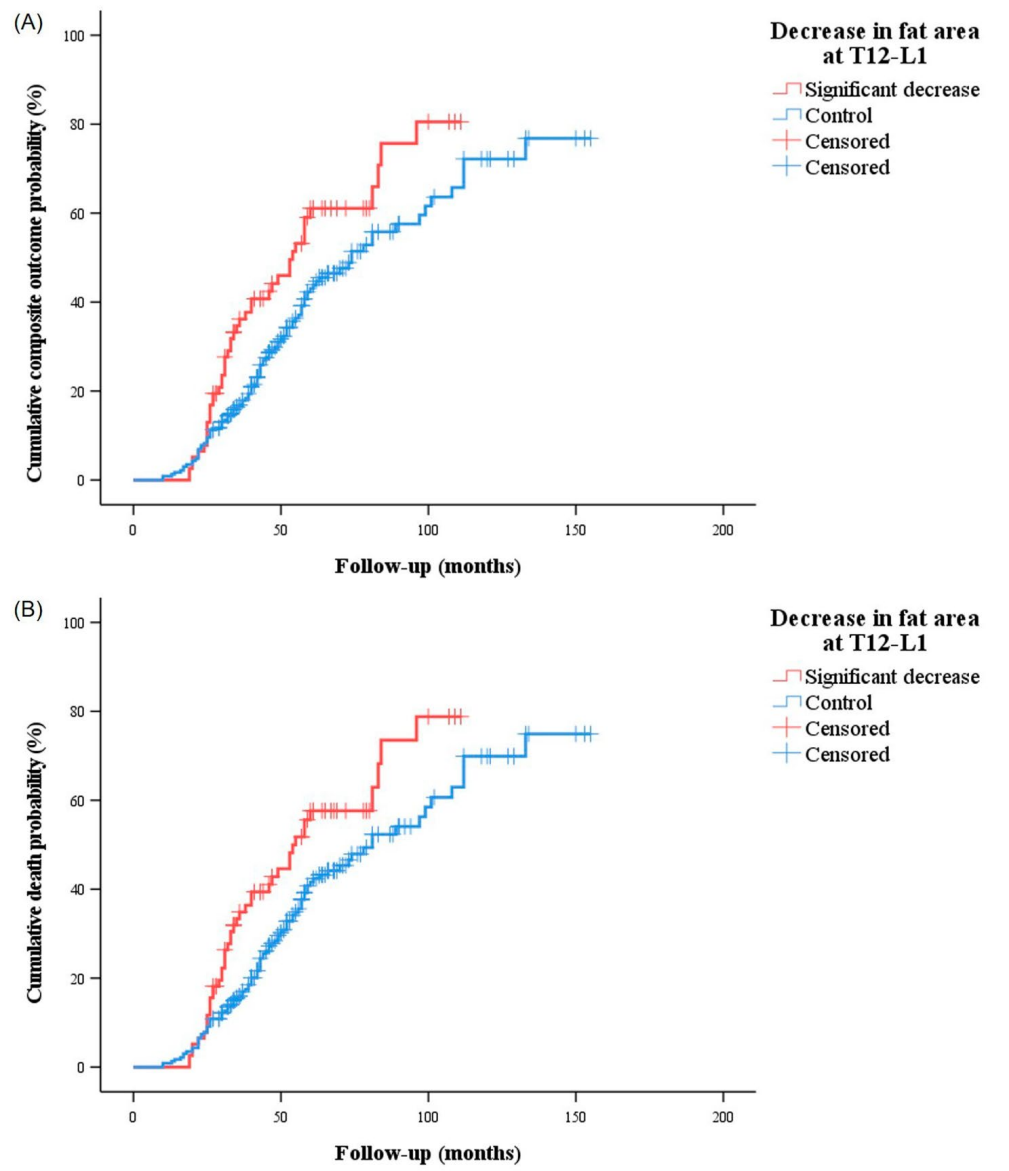
(**A**) Kaplan-Meier curve for the composite outcome with the log-rank test. Patients with a ≥ 52.3 cm^2^ decrease of fat area at T12-L1 during the first year post-diagnosis disease course were more likely to have the composite outcome than their counterparts (58.4% versus 43.9%, *P* = .007). (**B**) Kaplan Meier curve for death with the log-rank test. Patients with a ≥ 52.3 cm^2^ decrease in fat area at T12-L1 during the first year post-diagnosis had a higher frequency of death than those without it (58.3% versus 44.2%, *P* = .008)

As shown by the Kaplan-Meier curves, patients with a <-52.3 cm^2^ fat area change had higher frequencies of the composite outcome (58.4% vs. 43.9%, *P* = .007) and death (58.3% vs. 44.2%, *P* = .008) than their counterparts (Fig. 5).

### Subgroup analyses

Among 185 patients with underweight or normal baseline BMI, the lowest quartile of fat area decrease (threshold: -40.2 cm^2^) was associated with significantly higher rates of the composite outcomes in all Cox regression models of baseline clinical-radiological variables (adjusted HR: 1.704; 95% CI: 1.042, 2.787, *P* = .034), baseline clinical-radiological variables including GAP score (adjusted HR: 1.652; 95% CI: 1.027, 2.659, *P* = .039), and variables obtained at one-year follow-ups (adjusted HR: 1.954; 95% CI: 1.224, 3.119, *P* = .005) (Table S3). The results for death alone as an outcome are described in Table S4.

However, in the 122 patients with overweight or obese BMI at the baseline, fat area change (threshold: -75.4 cm^2^) was not associated with the composite outcome (*P* = .518) or death (*P* = .646) (Table S5 and Table S6).

## Discussion

The clinical course of idiopathic pulmonary fibrosis (IPF) and the rate of disease progression are unpredictable and heterogeneous [1]. To stratify the prognosis of patients with IPF, several prognostic factors, including pulmonary function test (PFT) results, fibrosis extent on CT images, mean pulmonary arterial pressure, aging, and cigarette smoke, have been developed [1, 6, 13, 30, 32, 33]. Although weight loss and low baseline BMI have been identified as poor prognostic factors in IPF patients [11, 14, 15, 17, 18, 20–22], the prognostic role of change in body fat remains unclear because of difficulties in measuring body composition. While body composition parameters obtained from bioelectric impedance and dual-energy X-ray absorptiometry have shown correlations with lung function, health-related quality of life, and survival in IPF and other fibrotic interstitial lung diseases, their focus has primarily been on fat-free mass or skeletal muscle, rather than body fat [34–36]. We addressed this issue in 307 IPF patients, using deep learning applied to chest CT images. A change in the fat area showed a positive correlation with a decline in FVC (*r* = .122; *P* = .032). A <-52.3 cm^2^ change in the fat area was a significant prognostic factor for the composite outcome and death alone after adjusting for various baseline clinical-radiological variables and variables obtained at one-year follow-ups, including PFT results and IPF extent. However, a change in BMI during the first year was not a significant prognostic factor. In subgroup analyses, a decrease in the fat area was a prominent prognostic factor in patients with an underweight to normal baseline BMI.

CT imaging is a fundamental aspect for IPF evaluation, including diagnosis, longitudinal follow-up, and prognostication [37]. Although IPF extent on CT images is a well-known prognostic factor in patients with IPF [38], other quantitative prognostic information can be obtained using CT images. Body composition analysis results are a prime example [23], and in this perspective, we explored the prognostic value of the change in body fat derived from CT images. Given the prognostic value of body fat change based on routinely performed CT images was proven in this study, patients’ prognostic and nutritional information can be obtained without additional radiation exposure or medical expenditure.

The results of this study are in line with previous studies assessing the prognostic value of weight loss and its association with FVC decline in IPF patients. Specifically, significant weight loss, defined as ≥ 5% of baseline weight, was an independent predictor of mortality [11, 12, 15, 18–22, 39], acute exacerbation [15], and hospitalization [16] in IPF patients. Regarding PFT results, a greater FVC decline in IPF patients was reported to be associated with significant weight loss and low baseline BMI [11, 14, 15, 20, 21]. Considering these results, a decrease in body fat is speculated to affect FVC decline, a key prognostic factor in IPF patients. Nevertheless, as demonstrated in the multivariable regression analyses, a considerable change in body fat (e.g., -52.3 cm^2^ in our study) was an independent prognostic factor after adjusting for declines in PFT results and IPF extent. In summary, we speculate that a decrease in body fat contributes to a poor prognosis in IPF patients both as an independent prognostic factor and by negatively affecting PFT results. While the association remains unclear, the decrease in body fat might be attributed to the pulmonary disease itself. The progression of IPF and the increase in dyspnea can impact the patient’s nutritional status, leading to greater malnutrition. Moreover, these nutritional deterioration might exacerbate dyspnea, creating a vicious cycle that ultimately contributes to adverse outcomes, potentially leading to death [4, 16, 40–42].

Segmenting and measuring body fat directly from CT images, using deep learning techniques, can better reflect the relationship between individuals’ metabolic status and prognosis than using weight and BMI, which represent a simple summation of the entire body weight, including skeletal muscle, bone, and organs [23, 43]. In our study, while baseline BMI was a significant prognostic factor in line with previous studies [14–17], the change in BMI during the first year was not; in contrast, the change in fat area during the first year consistently demonstrated prognostic value. We speculate that because BMI reflects other organs, which rarely change over the first year, the change in BMI during the early follow-up period may not hold prognostic value. In addition, the prognostic value of BMI decrease might be prominent in patients with high BMI. Indeed, baseline BMI were higher in prior study proving prognostic value of BMI decrease (28.2 kg/m^2^ [12] and 29.9 km/m^2^ [19]) than that of our study sample (24.5 kg/m^2^). In line with this, our subgroup analysis results showed that a decline in BMI had a negative prognostic effect only in overweight to obese patients. In contrast, since only fat can be extracted from CT images, it allows for a more thorough exploration of prognostic implications.

Several limitations of this study should be considered. First, this was a retrospective study, and many patients were excluded because they lacked available baseline or one-year follow-up CT or PFT results. Despite buffering for 3 months to alleviate variability in the patients’ follow-up strategy, we still did not sufficiently cover the heterogeneous follow-up strategies in many patients. However, extending the buffering period beyond 3 months might compromise the original purpose of this study. Second, heterogeneity in CT vendors, machines, and reconstruction protocols (e.g., reconstruction kernel) might have affected the quantification results. To mitigate this limitation, we used standard-kernel and thin-slice thickness images as much as possible because these reconstruction protocols were aligned with those of the training datasets of the body composition analysis software for this study. Future research using unified CT protocols or deep learning-based kernel conversion may be needed. Third, our study did not validate the threshold of significant fat change (-52.3 cm^2^) in an independent IPF cohort. Moreover, although we demonstrated that the association pattern between fat change during the first year and each outcome was not linear, the inconsistency in the prognostic significance of fat area change when treated as a continuous variable raises concerns about the robustness of our results. Therefore, additional validation studies with independent datasets are necessary to prove the prognostic implication of this threshold. Fourth, unlike our results, several studies have reported low baseline skeletal muscle and its decrease during follow-up as an adverse prognostic factor in IPF patients [44–46]. Differences in measurement methods (e.g., measurement of erector muscle versus all muscles) and the slight change in skeletal muscle quantification results in our study (e.g., a change of -0.1 cm^2^ during the first year) might explain this discrepancy. Further multi-center studies with consistent measurement methods will be needed to investigate the prognostic value of skeletal muscle quantification in IPF patients. Finally, although we analyzed one-year follow-up CT images, additional serial CT images of shorter intervals or beyond one year could provide more information about body composition changes and their prognostic value in IPF patients.

## Conclusion

In conclusion, a ≥ 52.3 cm^2^ decrease in fat area, automatically measured using deep learning technique, at T12-L1 in the first year post-diagnosis was an independent poor prognostic factor in patients with IPF. A management strategy to maintain body fat might improve outcomes in IPF patients.

### Electronic supplementary material

Below is the link to the electronic supplementary material.

Supplementary Material 1

## Data Availability

The datasets used and/or analyzed during the current study are available from the corresponding authors on reasonable request.

## Abbreviations

IPF: Idiopathic pulmonary fibrosis
FVC: Forced vital capacity
DL_CO_: Diffusing capacity of carbon monoxide
GAP: Gender, age, and physiology
BMI: Body mass index
PFT: Pulmonary function tests
