# Supplementary material for: Association between body fat decrease during the first year after diagnosis and the prognosis of idiopathic pulmonary fibrosis: CT-based body composition analysis
Source: Respir Res. 2024 Feb 28;25:103. doi: 10.1186/s12931-024-02712-6 (PMC10903156; doi:10.1186/s12931-024-02712-6)

**Additional File**

Appendix S1. Categorization method of the study cohort.

The association pattern between change in the fat area at T12-L1 during the first year after diagnosis and each of event (composite outcome and death) was assessed using natural cubic splines in a Cox regression model. As the association did not demonstrate a linear trend in the log scale of hazard ratio (HR) (Fig S1), we did not use the continuous variable. Instead, we identified that the x-axis point depicting the change in the fat area, where the HR's slope shifted notably, closely aligned with the threshold (-52.3 cm^2^) that divided patients into the lowest quartile based on their fat area change. Consequently, we used a categorical variable with this threshold.

Table S1. Univariable and multivariable Cox regression analysis for the composite outcome (body composition variables as continuous)

|  | Univariable analysis | | Multivariable analysis (model 1) | | Multivariable analysis (model 2) | | Multivariable analysis (model 3) | |
| --- | --- | --- | --- | --- | --- | --- | --- | --- |
| Variables | Hazard ratio | *P* value | Hazard ratio | *P* value | Hazard ratio | *P* value | Hazard ratio | *P* value |
| Age (year) | 1.027 (1.006, 1.047) | .009 | 1.050 (1.029, 1.073) | <.001 |  |  |  |  |
| Sex (reference: female) | 1.368 (0.921, 2.031) | .120 | 1.872 (1.037, 3.377) | .037 |  |  |  |  |
| BMI (kg/m^2^) | 0.955 (0.901, 1.013) | .125 | 0.875 (0.821, 0.933) | <.001 | 0.872 (0.816, 0.930) | <.001 |  |  |
| Change in BMI during the first year post-diagnosis (kg/m^2^) | 0.919 (0.801, 1.055) | .229 |  |  |  |  | 0.950 (0.860, 1.151) | .950 |
| Smoking history (reference: never smoker) | 1.328 (0.932, 1.892) | .117 | 1.219 (0.734, 2.022) | .444 | 1.228 (0.849, 1.776) | .275 |  |  |
| Antifibrotics (reference: no use) | 1.021 (0.703, 1.482) | .914 |  |  |  |  |  |  |
| Baseline GAP score | 1.662 (1.418, 1.948) | <.001 |  |  | 1.565 (1.324, 1.851) | <.001 |  |  |
| Baseline FVC (% predicted) | 0.979 (0.968, 0.990) | <.001 | 0.987 (0.975, 0.999) | .038 |  |  |  |  |
| Baseline DL_CO_ (% predicted) | 0.978 (0.968, 0.987) | <.001 | 0.975 (0.963, 0.987) | <.001 |  |  |  |  |
| FVC decline ≥ 5% during the first year post-diagnosis (% predicted) (reference: FVC decline < 5%) | 2.244 (1.615, 3.118) | <.001 |  |  |  |  | 2.201 (1.573, 3.080) | <.001 |
| DL_CO_ decline ≥ 10% during the first year post-diagnosis (% predicted) (reference: DL_CO_ decline < 10%) | 2.261 (1.577, 3.241) | <.001 |  |  |  |  | 2.368 (1.646, 3.406) | <.001 |
| Baseline pulmonary artery diameter (mm) | 1.080 (1.037, 1.125) | <.001 | 1.083 (1.035, 1.134) | .001 | 1.092 (1.041, 1.145) | <.001 |  |  |
| IPF extent on baseline CT (%) | 1.023 (1.011, 1.034) | <.001 | 1.007 (0.992, 1.023) | .352 | 1.018 (1.004, 1.031) | .009 |  |  |
| Muscle area at T12-L1 on baseline CT (cm^2^) (per 1 cm^2^ increase) | 1.000 (0.992, 1.007) | .922 |  |  |  |  |  |  |
| Change in muscle area at T12-L1 during the first year post-diagnosis (cm^2^) (per 1 cm^2^ increase) | 0.996 (0.983, 1.009) | .527 |  |  |  |  |  |  |
| Fat area at T12-L1 on baseline CT (cm^2^) (per 1 cm^2^ increase) | 1.001 (0.999, 1.003) | .391 |  |  |  |  |  |  |
| Change in the fat area at T12-L1 during the first year post-diagnosis (cm^2^) (per 1 cm^2^ increase) | 0.997 (0.995, 1.000) | .028 | 0.997 (0.994, 1.000) | .072 | 0.998 (0.995, 1.001) | .121 | 0.997 (0.995, 1.000) | .055 |

BMI: body mass index; GAP: gender, age, and physiologic variables; FVC: forced vital capacity; DL_CO_: diffusing capacity of carbon monoxide; IPF: idiopathic pulmonary fibrosis

Model 1 is with baseline clinical-radiological variables; Model 2 is with baseline clinical-radiological variables including GAP score; Model 3 is with variables obtained at 1-year follow-ups.

The composite outcome was the occurrence of death or lung transplantation.

Multivariable Cox proportional hazard regression analysis was performed with variables that had *P*-values <.2 in the univariable analysis and BMI change since this factor was reported as a poor prognostic factor in prior study.

Table S2. Univariable and multivariable Cox regression analysis for death (body composition variables as continuous)

|  | Univariable analysis | | Multivariable analysis (model 1) | | Multivariable analysis (model 2) | | Multivariable analysis (model 3) | |
| --- | --- | --- | --- | --- | --- | --- | --- | --- |
| Variables | Hazard ratio | *P* value | Hazard ratio | *P* value | Hazard ratio | *P* value | Hazard ratio | *P* value |
| Age (year) | 1.039 (1.018, 1.060) | <.001 | 1.063 (1.040, 1.086) | <.001 |  |  |  |  |
| Sex (reference: female) | 1.399 (0.931, 2.102) | .107 | 1.878 (1.025, 3.441) | .041 |  |  |  |  |
| BMI (kg/m^2^) | 0.966 (0.910, 1.024) | .244 | .891 (0.835, 0.950) | <.001 | 0.884 (0.827, 0.944) | <.001 |  |  |
| Change in BMI during the first year post-diagnosis (kg/m^2^) | 0.927 (0.805, 1.067) | .290 |  |  |  |  | 1.013 (0.873, 1.174) | .868 |
| Smoking history (reference: never smoker) | 1.311 (0.912, 1.884) | .143 | 1.197 (0.714, 2.006) | .496 | 1.178 (0.809, 1.715) | .393 |  |  |
| Antifibrotics (reference: no use) | 0.985 (0.674, 1.439) | .937 |  |  |  |  |  |  |
| Baseline GAP score | 1.747 (1.483, 2.058) | <.001 |  |  | 1.661 (1.398, 1.975) | <.001 |  |  |
| Baseline FVC (% predicted) | 0.981 (0.970, 0.992) | .001 | 0.985 (0.973, 0.998) | .020 |  |  |  |  |
| Baseline DL_CO_ (% predicted) | 0.981 (0.971, 0.991) | <.001 | 0.978 (0.967, 0.990) | <.001 |  |  |  |  |
| FVC decline ≥ 5% during the first year post-diagnosis (% predicted) (reference: FVC decline < 5%) | 2.113 (1.507, 2.962) | <.001 |  |  |  |  | 2.067 (1.462, 2.921) | <.001 |
| DL_CO_ decline ≥ 10% during the first year post-diagnosis (% predicted) (reference: DL_CO_ decline < 10%) | 2.179 (1.506, 3.151) | <.001 |  |  |  |  | 2.234 (1.541, 3.239) | <.001 |
| Baseline pulmonary artery diameter (mm) | 1.080 (1.036, 1.126) | <.001 | 1.081 (1.031, 1.133) | .001 | 1.087 (1.036, 1.141) | .001 |  |  |
| IPF extent on baseline CT (%) | 1.023 (1.011, 1.035) | <.001 | 1.010 (0.995, 1.026) | .197 | 1.661 (1.398, 1.975) | <.001 |  |  |
| Muscle area at T12-L1 on baseline CT (cm^2^) (per 1 cm^2^ increase) | 0.998 (0.990, 1.006) | .596 |  |  |  |  |  |  |
| Change in muscle area at T12-L1 during the first year post-diagnosis (cm^2^) (per 1 cm^2^ increase) | 0.998 (0.986, 1.011) | .808 |  |  |  |  |  |  |
| Fat area at T12-L1 on baseline CT (cm^2^) (per 1 cm^2^ increase) | 1.001 (0.999,1.003) | .261 |  |  |  |  |  |  |
| Change in the fat area at T12-L1 during the first year post-diagnosis (cm^2^) (per 1 cm^2^ increase) | 0.997 (0.995, 1.000) | .038 | 0.997 (0.994, 1.000) | .077 | 0.998 (0.995, 1.001) | .152 | 0.997 (0.995, 1.000) | .072 |

BMI: body mass index; GAP: gender, age, and physiologic variables; FVC: forced vital capacity; DL_CO_: diffusing capacity of carbon monoxide; IPF: idiopathic pulmonary fibrosis

Model 1 is with baseline clinical-radiological variables; Model 2 is with baseline clinical-radiological variables including GAP score; Model 3 is with variables obtained at 1-year follow-ups.

Multivariable Cox proportional hazard regression analysis was performed with variables with *P*-values <.2 in the univariable analysis and BMI change since this factor was reported as a poor prognostic factor in prior study.

Table S3. Univariable and multivariable Cox regression analysis for composite outcome in patients with underweight to normal baseline BMI (<25 kg/m^2^)

|  | Univariable analysis | | Multivariable analysis (model 1) | | Multivariable analysis (model 2) | | Multivariable analysis (model 3) | |
| --- | --- | --- | --- | --- | --- | --- | --- | --- |
| Variables | Hazard ratio | *P* value | Hazard ratio | *P* value | Hazard ratio | *P* value | Hazard ratio | *P* value |
| Age (year) | 1.029 (1.003, 1.055) | .028 | 1.051 (1.021, 1.082) | .001 |  |  |  |  |
| Sex (reference: female) | 1.518 (0.918, 2.508) | .104 | 1.572 (0.729, 3.391) | .248 |  |  |  |  |
| BMI (kg/m^2^) | 0.815 (0.724, 0.917) | .001 | 0.8 (0.707, 0.906) | <.001 | 0.766 (0.680, 0.864) | <.001 |  |  |
| Change in BMI during the first year post-diagnosis (kg/m^2^) | 0.972 (0.817, 1.157) | .751 |  |  |  |  | 1.076 (0.906, 1.278) | .404 |
| Smoking history (reference: never smoker) | 1.677 (1.059, 2.656) | .028 | 1.498 (0.771, 2.908) | .233 | 1.431 (0.885, 2.314) | .144 |  |  |
| Antifibrotics (reference: no use) | 1.291 (0.784, 2.125) | .316 |  |  |  |  |  |  |
| Baseline GAP score | 1.685 (1.365, 2.081) | <.001 |  |  | 1.547 (1.225, 1.954) | <.001 |  |  |
| Baseline FVC (% predicted) | 0.976 (0.963, 0.989) | <.001 | 0.983 (0.967, 0.999) | .036 |  |  |  |  |
| Baseline DL_CO_ (% predicted) | 0.98 (0.968, 0.992) | .001 | 0.979 (0.963, 0.995) | .01 |  |  |  |  |
| FVC decline ≥ 5% during the first year post-diagnosis (% predicted) (reference: FVC decline < 5%) | 2.283 (1.498, 3.480) | <.001 |  |  |  |  | 2.044 (1.316, 3.173) | .001 |
| DL_CO_ decline ≥ 10% during the first year post-diagnosis (% predicted) (reference: DL_CO_ decline < 10%) | 2.887 (1.867, 4.463) | <.001 |  |  |  |  | 2.799 (1.799, 4.356) | <.001 |
| Baseline pulmonary artery diameter (mm) | 1.117 (1.054, 1.184) | <.001 | 1.071 (1.007, 1.139) | .028 | 1.093 (1.029, 1.161) | .004 |  |  |
| IPF extent on baseline CT (%) | 1.043 (1.023, 1.064) | <.001 | 1.011 (0.985, 1.037) | .417 | 1.022 (0.999, 1.046) | .061 |  |  |
| Muscle area at T12-L1 on baseline CT (cm^2^) (reference: upper three quartiles) | 0.956 (0.594, 1.540) | .854 |  |  |  |  |  |  |
| Change in muscle area at T12-L1 during the first year post-diagnosis (cm^2^) (reference: upper three quartiles) | 1.286 (0.790, 2.093) | .312 |  |  |  |  |  |  |
| Fat area at T12-L1 on baseline CT (cm^2^) (reference: upper three quartiles) | 1.284 (0.807, 2.045) | .291 |  |  |  |  |  |  |
| Change in the fat area at T12-L1 during the first year post-diagnosis (cm^2^) (reference: upper three quartiles) | 2.048 (1.304, 3.214) | .002 | 1.704 (1.042, 2.787) | .034 | 1.652 (1.027, 2.659) | .039 | 1.954 (1.224, 3.119) | .005 |

BMI: body mass index; GAP: gender, age, and physiologic variables; FVC: forced vital capacity; DL_CO_: diffusing capacity of carbon monoxide; IPF: idiopathic pulmonary fibrosis

Model 1 is with baseline clinical-radiological variables; Model 2 is with baseline clinical-radiological variables including GAP score; Model 3 is with variables obtained at 1-year follow-ups.

The composite outcome was the occurrence of death or lung transplantation.

Multivariable Cox proportional hazard regression analysis was performed with variables that had *P*-values <.2 in the univariable analysis and BMI change since this factor was reported as a poor prognostic factor in prior study.

Table S4. Univariable and multivariable Cox regression analysis for death in patients with underweight to normal baseline BMI (<25 kg/m^2^)

|  | Univariable analysis | | Multivariable analysis (model 1) | | Multivariable analysis (model 2) | | Multivariable analysis (model 3) | |
| --- | --- | --- | --- | --- | --- | --- | --- | --- |
| Variables | Hazard ratio | *P* value | Hazard ratio | *P* value | Hazard ratio | *P* value | Hazard ratio | *P* value |
| Age (year) | 1.043 (1.016, 1.071) | .002 | 1.067 (1.035, 1.1) | <.001 |  |  |  |  |
| Sex (reference: female) | 1.625 (0.960, 2.750) | .071 | 1.514 (0.685, 3.346) | .305 |  |  |  |  |
| BMI (kg/m^2^) | 0.837 (0.740, 0.947) | .005 | 0.835 (0.735, 0.949) | .006 | 0.791 (0.699, 0.896) | <.001 |  |  |
| Change in BMI during the first year post-diagnosis (kg/m^2^) | 0.985 (0.824, 1.178) | .871 |  |  |  |  | 1.112 (0.933, 1.326) | .236 |
| Smoking history (reference: never smoker) | 1.707 (1.060, 2.748) | .028 | 1.563 (0.789, 3.097) | .2 | 1.431 (0.872, 2.349) | .156 |  |  |
| Antifibrotics (reference: no use) | 1.277 (0.765, 2.131) | .350 |  |  |  |  |  |  |
| Baseline GAP score | 1.814 (1.455, 2.261) | <.001 |  |  | 1.690 (1.326, 2.155) | <.001 |  |  |
| Baseline FVC (% predicted) | 0.978 (0.964, 0.992) | .002 | 0.979 (0.963, 0.995) | .01 |  |  |  |  |
| Baseline DL_CO_ (% predicted) | 0.985 (0.973, 0.997) | .017 | 0.983 (0.968, 0.999) | .038 |  |  |  |  |
| FVC decline ≥ 5% during the first year post-diagnosis (% predicted) (reference: FVC decline < 5%) | 2.162 (1.401, 3.337) | .000 |  |  |  |  | 1.962 (1.242, 3.098) | .004 |
| DL_CO_ decline ≥ 10% during the first year post-diagnosis (% predicted) (reference: DL_CO_ decline < 10%) | 2.704 (1.727, 4.235) | .000 |  |  |  |  | 2.576 (1.636, 4.057) | <.001 |
| Baseline pulmonary artery diameter (mm) | 1.111 (1.046, 1.180) | .001 | 1.061 (0.996, 1.13) | .068 | 1.081 (1.016, 1.150) | .014 |  |  |
| IPF extent on baseline CT (%) | 1.045 (1.023, 1.066) | .000 | 1.016 (0.99, 1.042) | .226 | 1.024 (1.000, 1.049) | .051 |  |  |
| Muscle area at T12-L1 on baseline CT (cm^2^) (reference: upper three quartiles) | 0.910 (0.554, 1.494) | .708 |  |  |  |  |  |  |
| Change in muscle area at T12-L1 during the first year post-diagnosis (cm^2^) (reference: upper three quartiles) | 1.077 (0.642, 1.805) | .779 |  |  |  |  |  |  |
| Fat area at T12-L1 on baseline CT (cm^2^) (reference: upper three quartiles) | 1.113 (0.683, 1.816) | .667 |  |  |  |  |  |  |
| Change in the fat area at T12-L1 during the first year post-diagnosis (cm^2^) (reference: upper three quartiles) | 1.985 (1.246, 3.162) | .004 | 1.646 (0.988, 2.742) | .055 | 1.593 (0.977, 2.599) | .062 | 1.863 (1.144, 3.034) | .012 |

BMI: body mass index; GAP: gender, age, and physiologic variables; FVC: forced vital capacity; DL_CO_: diffusing capacity of carbon monoxide; IPF: idiopathic pulmonary fibrosis

Model 1 is with baseline clinical-radiological variables; Model 2 is with baseline clinical-radiological variables including GAP score; Model 3 is with variables obtained at 1-year follow-ups.

Multivariable Cox proportional hazard regression analysis was performed with variables with *P*-values <.2 in the univariable analysis and BMI change since this factor was reported as a poor prognostic factor in prior study.

Table S5. Univariable and multivariable Cox regression analysis for composite outcome in patients with overweight to obese baseline BMI (≥25 kg/m^2^)

|  | Univariable analysis | | Multivariable analysis (Baseline information) | | Multivariable analysis (Baseline GAP) | | Multivariable analysis (F/U information) | |
| --- | --- | --- | --- | --- | --- | --- | --- | --- |
| Variables | Hazard ratio | *P* value | Hazard ratio | *P* value | Hazard ratio | *P* value | Hazard ratio | *P* value |
| Age (year) | 1.019 (0.987, 1.052) | .245 |  |  |  |  |  |  |
| Sex (reference: female) | 1.177 (0.619, 2.239) | .619 |  |  |  |  |  |  |
| BMI (kg/m^2^) | 0.980 (0.855, 1.124) | .775 | 0.897 (0.773, 1.042) | .156 | 0.962 (0.831, 1.113) | .599 |  |  |
| Change in BMI during the first year post-diagnosis (kg/m^2^) | 0.778 (0.617, 0.981) | .034 |  |  |  |  | 0.775 (0.610, 0.984) | .036 |
| Smoking history (reference: never smoker) | 0.855 (0.489, 1.496) | .583 |  |  |  |  |  |  |
| Antifibrotics (reference: no use) | 0.695 (0.396, 1.220) | .205 |  |  |  |  |  |  |
| Baseline GAP score | 1.641 (1.277, 2.108) | <.001 |  |  | 1.638 (1.268, 2.116) | <.001 |  |  |
| Baseline FVC (% predicted) | 0.986 (0.967, 1.005) | .138 | 0.997 (0.978, 1.016) | .760 |  |  |  |  |
| Baseline DL_CO_ (% predicted) | 0.972 (0.955, 0.989) | .001 | 0.972 (0.953, 0.992) | .006 |  |  |  |  |
| FVC decline ≥ 5% during the first year post-diagnosis (% predicted) (reference: FVC decline < 5%) | 2.625 (1.497, 4.603) | .001 |  |  |  |  | 2.612 (1.489, 4.583) | .001 |
| DL_CO_ decline ≥ 10% during the first year post-diagnosis (% predicted) (reference: DL_CO_ decline < 10%) | 1.269 (0.621, 2.591) | .514 |  |  |  |  |  |  |
| Baseline pulmonary artery diameter (mm) | 1.049 (0.979, 1.124) | .177 | 1.039 (0.968, 1.115) | .290 | 1.068 (0.988, 1.154) | .099 |  |  |
| IPF extent on baseline CT (%) | 1.014 (0.998, 1.031) | .084 | 1.001 (0.981, 1.020) | .946 | 1.011 (0.994, 1.029) | .207 |  |  |
| Muscle area at T12-L1 on baseline CT (cm^2^) (reference: upper three quartiles) | 0.957 (0.523, 1.752) | .887 |  |  |  |  |  |  |
| Change in muscle area at T12-L1 during the first year post-diagnosis (cm^2^) (reference: upper three quartiles) | 1.008 (0.557, 1.824) | .980 |  |  |  |  |  |  |
| Fat area at T12-L1 on baseline CT (cm^2^) (reference: upper three quartiles) | 0.722 (0.379, 1.374) | .321 |  |  |  |  |  |  |
| Change in the fat area at T12-L1 during the first year post-diagnosis (cm^2^) (reference: upper three quartiles) | 1.261 (0.673, 2.196) | .518 |  |  |  |  |  |  |

BMI: body mass index; GAP: gender, age, and physiologic variables; FVC: forced vital capacity; DL_CO_: diffusing capacity of carbon monoxide; IPF: idiopathic pulmonary fibrosis

Model 1 is with baseline clinical-radiological variables; Model 2 is with baseline clinical-radiological variables including GAP score; Model 3 is with variables obtained at 1-year follow-ups.

The composite outcome was the occurrence of death or lung transplantation.

Multivariable Cox proportional hazard regression analysis was performed with variables that had *P*-values <.2 in the univariable analysis and BMI since this factor was reported as a poor prognostic factor in prior study.

.

Table S6. Univariable and multivariable Cox regression analysis for death in patients with overweight to obese baseline BMI (≥25 kg/m^2^)

|  | Univariable analysis | | Multivariable analysis (model 1) | | Multivariable analysis (model 2) | | Multivariable analysis (model 3) | |
| --- | --- | --- | --- | --- | --- | --- | --- | --- |
| Variables | Hazard ratio | *P* value | Hazard ratio | *P* value | Hazard ratio | *P* value | Hazard ratio | *P* value |
| Age (year) | 1.028 (0.995, 1.062) | .099 | 1.028 (0.991, 1.068) | .142 |  |  |  |  |
| Sex (reference: female) | 1.092 (0.573, 2.082) | .789 |  |  |  |  |  |  |
| BMI (kg/m^2^) | 0.964 (0.838, 1.110) | .613 | 0.891 (0.755, 1.053) | .176 | 0.935 (0.801, 1.090) | .388 |  |  |
| Change in BMI during the first year post-diagnosis (kg/m^2^) | 0.786 (0.621, 0.995) | .045 |  |  |  |  | 0.782 (0.614, 0.995) | .046 |
| Smoking history (reference: never smoker) | 0.798 (0.455, 1.402) | .433 |  |  |  |  |  |  |
| Antifibrotics (reference: no use) | 0.65 (0.369, 1.145) | .136 | 0.760 (0.396, .1.458) | .409 |  |  |  |  |
| Baseline GAP score | 1.674 (1.297, 2.161) | <.001 |  |  | 1.654 (1.271, 2.152) | <.001 |  |  |
| Baseline FVC (% predicted) | 0.987 (0.968, 1.006) | .166 | 0.992 (0.974, 1.012) | .438 |  |  |  |  |
| Baseline DL_CO_ (% predicted) | 0.857 (0.787, 0.932) | .000 | 0.976 (0.957, 0.995) | .014 |  |  |  |  |
| FVC decline ≥ 5% during the first year post-diagnosis (% predicted) (reference: FVC decline < 5%) | 2.373 (1.349, 4.172) | .003 |  |  |  |  | 2.372 (1.348, 4.174) | .003 |
| DL_CO_ decline ≥ 10% during the first year post-diagnosis (% predicted) (reference: DL_CO_ decline < 10%) | 1.354 (0.661, 2.772) | .408 |  |  |  |  |  |  |
| Baseline pulmonary artery diameter (mm) | 1.051 (0.979, 1.128) | .169 | 1.056 (0.981, 1.138) | .149 | 1.075 (0.992, 1.164) | .078 |  |  |
| IPF extent on baseline CT (%) | 1.014 (0.997, 1.031) | .104 | 1.002 (0.981, 1.023) | .882 | 1.009 (0.991, 1.028) | .327 |  |  |
| Muscle area at T12-L1 on baseline CT (cm^2^) (reference: upper three quartiles) | 1.013 (0.552, 1.860) | .967 |  |  |  |  |  |  |
| Change in muscle area at T12-L1 during the first year post-diagnosis (cm^2^) (reference: upper three quartiles) | 1.072 (0.590, 1.948) | .819 |  |  |  |  |  |  |
| Fat area at T12-L1 on baseline CT (cm^2^) (reference: upper three quartiles) | 0.768 (0.402, 1.466) | .423 |  |  |  |  |  |  |
| Change in the fat area at T12-L1 during the first year post-diagnosis (cm^2^) (reference: upper three quartiles) | 1.153 (0.627, 2.119) | .646 |  |  |  |  |  |  |

BMI: body mass index; GAP: gender, age, and physiologic variables; FVC: forced vital capacity; DL_CO_: diffusing capacity of carbon monoxide; IPF: idiopathic pulmonary fibrosis

Model 1 is with baseline clinical-radiological variables; Model 2 is with baseline clinical-radiological variables including GAP score; Model 3 is with variables obtained at 1-year follow-ups.

Multivariable Cox proportional hazard regression analysis was performed with variables that had *P*-values <.2 in the univariable analysis and BMI since this factor was reported as a poor prognostic factor in prior study.

Figure S1. Association pattern between change in the fat area at T12-L1 during the first year after diagnosis and each of event was assessed using natural cubic splines in a Cox regression model. (A) Composite outcome. (B) Death.


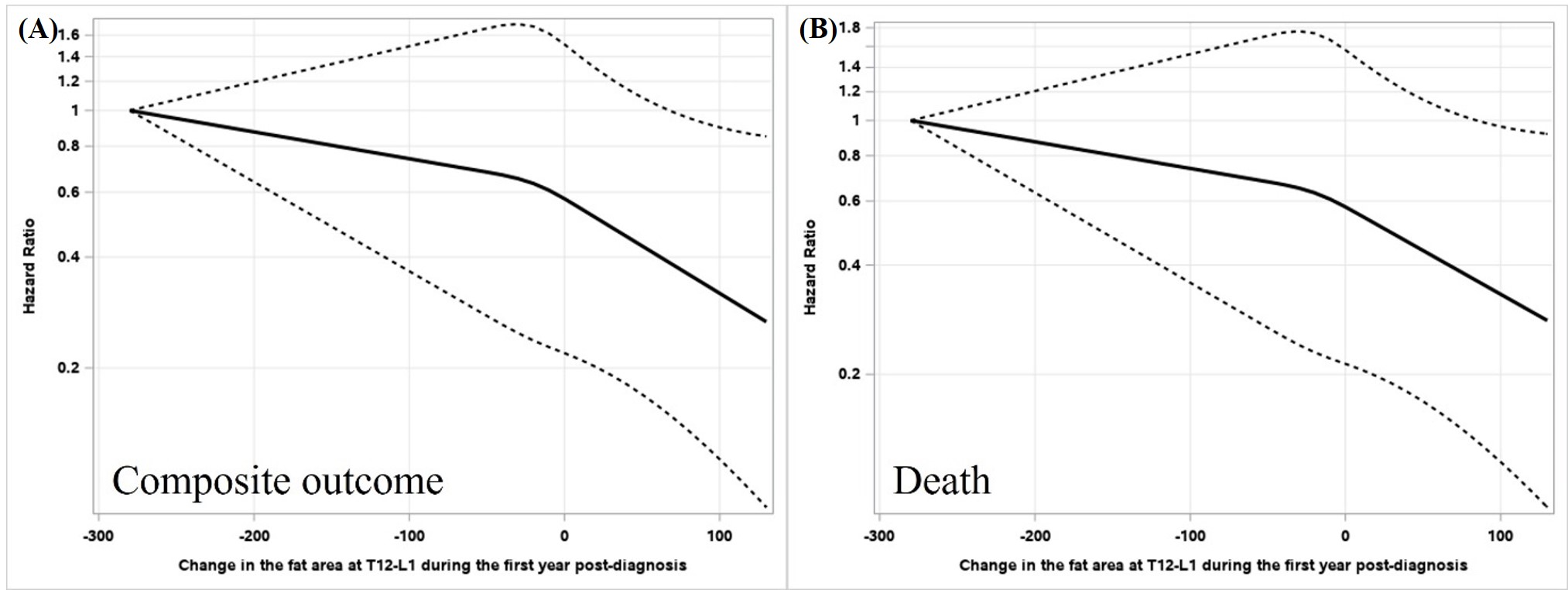


Figure S2. Plots of Spearman correlation coefficients between changes in the fat area at the level of T12-L1 and changes in body mass index during the first year after diagnosis (*r*=0.335; *P*<.001).


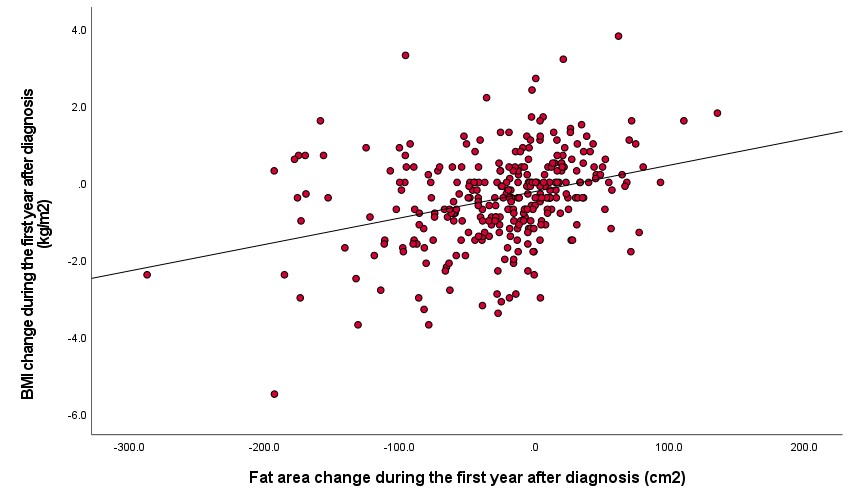

Supplement: Supplementary file 1 — Supplementary Material 1 [file 12931_2024_2712_MOESM1_ESM.docx]
